# Epigenetic regulation of NKG2D ligands is involved in exacerbated atherosclerosis development in Sirt6 heterozygous mice

**DOI:** 10.1038/srep23912

**Published:** 2016-04-05

**Authors:** Zhu-Qin Zhang, Si-Chong Ren, Ying Tan, Zuo-Zhi Li, Xiaoqiang Tang, Ting-Ting Wang, De-Long Hao, Xiang Zhao, Hou-Zao Chen, De-Pei Liu

**Affiliations:** 1 State Key Laboratory of Medical Molecular Biology, Department of Biochemistry and Molecular Biology, Institute of Basic Medical Sciences, Chinese Academy of Medical Sciences and Peking Union Medical College, No. 5 Dong Dan San Tiao, Beijing 100005, P.R. China

## Abstract

Sirt6 is a member of the class III histone deacetylase family which is associated with aging and longevity. Sirt6 deficient mice show an aging-like phenotype, while male transgenic mice of Sirt6 show increased longevity. Sirt6 acts as a tumor suppressor and deficiency of Sirt6 leads to cardiac hypertrophy and heart failure. Whether Sirt6 is involved in atherosclerosis development, the major cause of cardiovascular diseases, is unknown. We found that the expression of Sirt6 is lower in human atherosclerotic plaques than that in controls. When Sirt6^+/−^ApoE^−/−^ and ApoE^−/−^ mice are fed with high fat diet for 16 weeks, Sirt6^+/−^ApoE^−/−^ mice show increased plaque fromation and exhibit feature of plaque instability. Furthermore, Sirt6 downregulation increases expression of NKG2D ligands, which leads to increased cytokine expression. Blocking NKG2D ligand almost completely blocks this effect. Mechanistically, Sirt6 binds to promoters of NKG2D ligand genes and regulates the H3K9 and H3K56 acetylation levels.

Atherosclerosis, the major cause of cardiovascular diseases, is aging-associated disease caused by complex genetic and environmental factors. Immunity and inflammation are two key aspects for atherosclerosis development. Different immune cells, including macrophages, T cells, and NK cells, and multiple immune pathways, including adaptive and innate immunity, are involved in the process of atherosclerosis development^1^,^2^,^3^. Some inflammatory factors, such as TNF-α, IFN-γ and IL-1β, are pro-inflammatory and increase atherosclerotic plaque formation^4^,^5^. The immune and inflammation forms a complex network to regulate atherogenesis^6^.

Sirt6 is a member of the class III histone deacetylase family^7^. Sirt6 deficient mice show an aging-like phenotype^8^, while male transgenic mice of Sirt6 show increased longevity^9^. Tumor and cardiovascular diseases are aging associated diseases. Sirt6 acts as a tumor suppressor^10^. Sirt6 deficiency promotes the initiation of cancer^11^ and a low Sirt6 level is associated with poor clinical outcome in patients^12^. In cardiovascular system, Sirt6 knockout mice have been reported to develop increased cardiac hypertrophy and heart failure^13^. Whether Sirt6 directly affects atherosclerosis is unknown.

Here we found that Sirt6 expression level is decreased in athersclerotic plaques. Sirt6 heterozygosity exacerbates atherogenesis and shows feature of plaque instability. Epigenetic regulation of expression of NKG2D ligand, one important type of molecules in innate immunity, is involved in this process. Sirt6 heterozygosity shows increased NKG2D ligand expression, leading to NK cell activation and increased levels of inflammatory cytokines in NK cells. Blocking of NKG2D ligand-receptor interaction almost completely blocks the effect of Sirt6 heterozygosity. Mechanistically, Sirt6 regulates H3K9 and H3K56 acetylation levels of NKG2D ligand gene promoters.

## Results

### Sirt6 expression is downregulated in human atherosclerotic plaques

**Figure 1:**
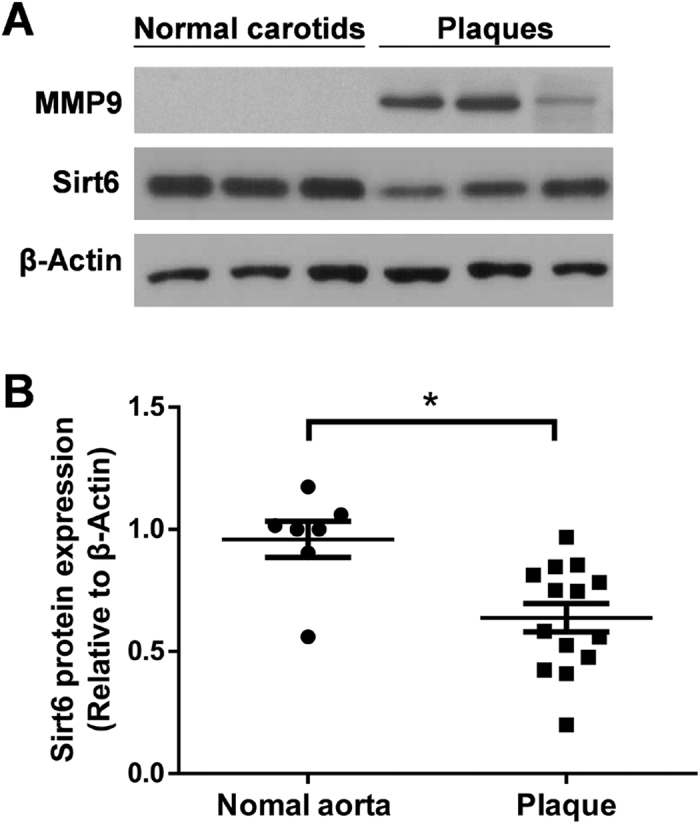
Sirt6 protein expression is lower in human carotid endarterectomy specimens than in normal carotids. (**A**) The Sirt6 protein level in human atherosclerotic plaques and normal carotids from controls was analyzed in representative western blot. MMP9 acts as a positive control. (**B**) Statistical analysis of the Sirt6 protein level relative to β-Actin in normal carotids (n = 7) and atherosclerotic plaques (n = 14) is shown. Each symbol represents a measurement from a single sample. Student’s t test was applied to calculate the P value. (*P < 0.05).

To determine whether Sirt6 is involved in atherosclerosis, we assessed the Sirt6 expression levels in atherosclerotic plaques from patients undergoing carotid endarterectomy and in carotid arteries of controls. The Sirt6 protein level in the carotid atherosclerotic plaques was lower than that in the normal carotid samples, as shown in a representative western blot (Fig. 1A). The band intensity for Sirt6 relative to β-actin was analyzed statistically for all samples (Fig. 1B). The lower expression of Sirt6 in the atherosclerotic plaques suggests that Sirt6 may be involved in atherosclerotic progression.

### Sirt6 heterozygosity exacerbates atherosclerosis development in ApoE^−/−^ mice

**Figure 2:**
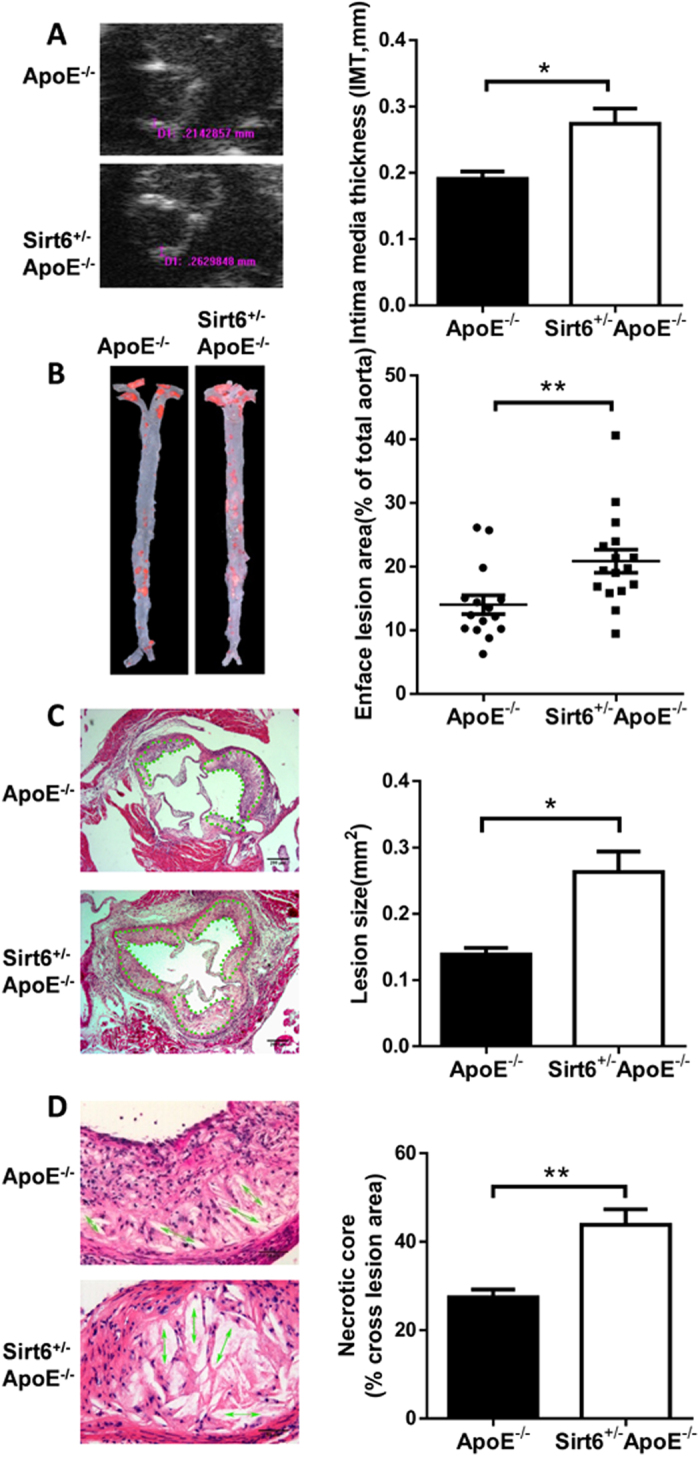
Sirt6 heterozygosity exacerbates atherosclerosis development. ApoE^−/−^ and Sirt6^+/−^ApoE^−/−^ mice were fed with Western diet for 16 weeks and plaque lesion was determined by different methods. (**A**) The intima media thickness (IMT) of the aortic root was measured by ultrasound biomicroscopy. ApoE^−/−^ group (n = 15), Sirt6^+/−^ApoE^−/−^ group (n = 13). (**B**) The plaque area in the aortas was determined by en face staining for lipids with Oil Red O. ApoE^−/−^ group (n = 15), Sirt6^+/−^ApoE^−/−^ group (n = 16). Quantification of the surface area relative to the total aorta was shown. Each symbol represents a measurement from a single mouse. (**C**) The atherosclerotic plaque lesion area of the aortic sinus was assessed by H&E staining. ApoE^−/−^ group (n = 10), Sirt6^+/−^ApoE^−/−^ group (n = 12). Dot lines indicate the area that was measured. (**D**) The area of the necrotic core of the atherosclerotic plaques was determined by H&E staining (n = 8 each group). Arrows indicate the field that was measured. Value was normalized to total cross lesion area. The statistical analysis is shown on the right side of each panel. Student’s t test was used to calculate the P value. (*P < 0.05, **P < 0.01).

The downregulation of Sirt6 in atherosclerotic plaques prompted us to study its roles in the development of atherosclerosis. Sirt6 homozygous knockout mice die at about one month of age^8^. We utilized heterozygous Sirt6 (Sirt6^+/−^) mice and wild-type mice to cross with ApoE^−/−^ mice, respectively (Supplementary Fig. 1). The offspring ApoE^−/−^ and Sirt6^+/−^ApoE^−/−^ mice were fed with normal chow diet (ND) or Western diet (WD) for 16 weeks. We found that when mice were fed with normal chow diet, both ApoE^−/−^ and Sirt6^+/−^ApoE^−/−^ mice showed no obvious atherosclerotic plaque (Supplementary Fig. 2). The atherosclerotic plaque area for ApoE^−/−^ and Sirt6^+/−^ApoE^−/−^ mice fed with Western diet was determined using several methods, as described below. Intima media thickness (IMT) is an important parameter of atherosclerotic plaque development, and it correlates with the severity of atherosclerotic plaques^14^. We measured the IMT of the aortic root. The IMT value was higher for the Sirt6^+/−^ApoE^−/−^ mice than for the ApoE^−/−^ mice (Fig. 2A). Similarly, the quantification of Oil Red O staining in aortas revealed that the Sirt6^+/−^ApoE^−/−^ had larger aortic plaque lesions (Fig. 2B). In addition, H&E staining of the aortic sinus revealed a significant increase in lesion size (Fig. 2C) in the Sirt6^+/−^ApoE^−/−^ mice. Necrotic core size in atherosclerotic plaques was larger in the Sirt6^+/−^ApoE^−/−^ mice compared with their ApoE^−/−^ littermates (Fig. 2D). Taken together, these results demonstrate that Sirt6 heterozygosity exacerbates atherosclerotic plaque development.

### Sirt6 heterozygosity enhances the feature of plaque instability in ApoE^−/−^ mice

**Figure 3:**
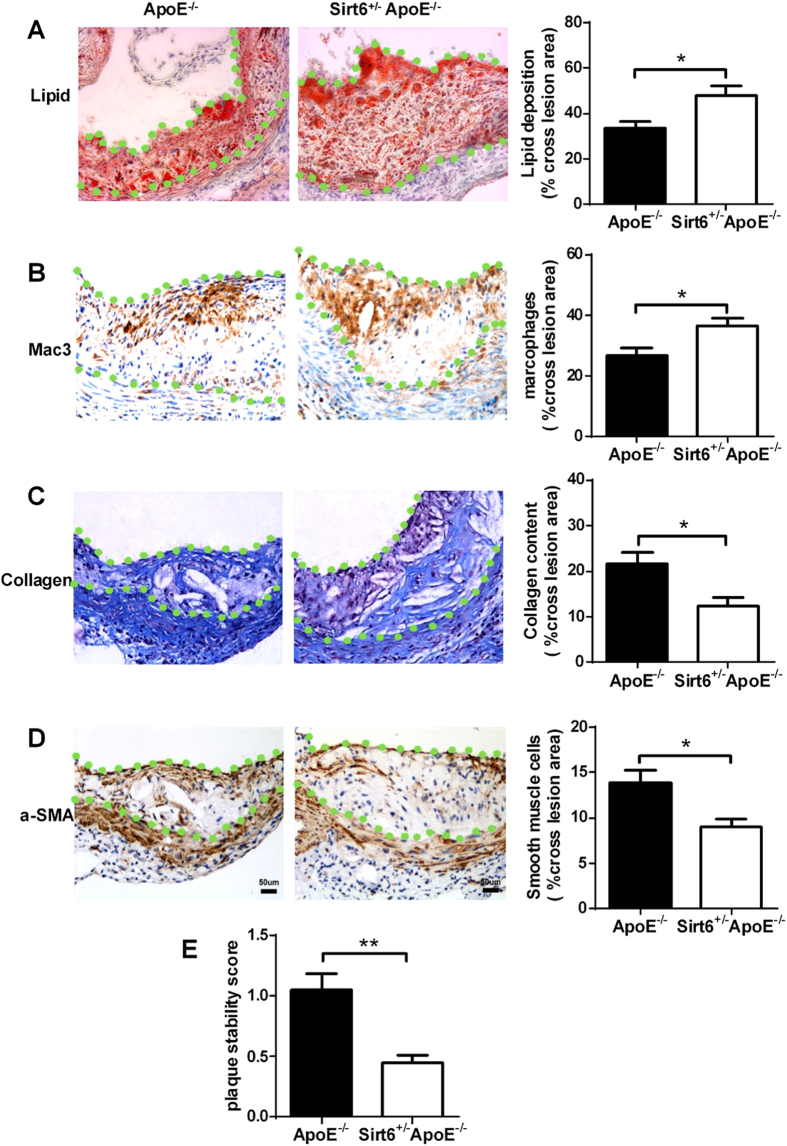
Sirt6 heterozygosity exhibits feature of more instable atherosclerotic plaque. ApoE^−/−^ and Sirt6^+/−^ApoE^−/−^ mice were fed with Western diet for 16 weeks and sections of mouse aortic roots were stained by different methods. (**A**) Oil Red O staining reveals the neutral lipid content. ApoE^−/−^ group (n = 8), Sirt6^+/−^ApoE^−/−^ group (n = 7). (**B**) Mac-3 staining shows the macrophage content. ApoE^−/−^ group (n = 8), Sirt6^+/−^ApoE^−/−^ group (n = 7). (**C**) Masson staining indicates the collagen content (n = 7 each group). (**D**) α-smooth muscle actin (α-SMA) staining indicates the smooth muscle cell content (n = 7 each group). The statistical analysis is shown on the right side. The total cross lesion areas are outlined with green dots and the positive signal was normalized to total cross lesion area in (**A**–**D**). (**E**) Plaque stability was calculated as the value of (SMC area + collagen area)/(macrophage area + lipid area)^34^ as described in Methods. Student’s t test was used to calculate the P value. (*P < 0.05, **P < 0.01).

Although plaque lesion size indicates the extent of atherosclerotic development, plaque stability is a more accurate predictor of plaque rupture and clinical events^15^,^16^. To investigate whether Sirt6 affects plaque stability, we evaluated several key parameters associated with plaque stability^16^. We found that the plaques in the Sirt6^+/−^ApoE^−/−^ mice contained more lipids than those in their ApoE^−/−^ littermates, as assessed by Oil red O staining of the aortic root (Fig. 3A). There was more macrophage infiltration in the atherosclerotic plaques in Sirt6^+/−^ApoE^−/−^ mice than in ApoE^−/−^ littermates (Fig. 3B). On the contrary, Masson’s trichrome staining revealed that the atherosclerotic plaque lesions in the Sirt6^+/−^ApoE^−/−^ mice contained lower percentage of collagen (Fig. 3C) and the number of α-smooth muscle actin-positive cells was lower in plaques of Sirt6^+/−^ApoE^−/−^ mice (Fig. 3D). Increases of lipid deposition, macrophage infiltration and decreases of smooth muscle cells, collagen content are feature that are attributed to a more instable plaque phenotype^15^. The Sirt6 heterozygous mice exhibited half of the plaque stability of the control animals, as calculated using the formula for plaque stability (Fig. 3E). Thus, these results indicate that Sirt6 heterozygosity promotes lesion instability in addition to exacerbating atherogenesis in ApoE^−/−^ mice.

### Sirt6 heterozygous mice exhibit increased expression of NKG2D ligands

For insight into mechanisms for Sirt6 heterozygosity exacerbated atherosclerosis, we investigated blood pressure (Supplementary Table 1), serum glucose and lipid level (Supplementary Table 2) and body weight (Supplementary Fig. 3) in ApoE^−/−^ mice and Sirt6^+/−^ApoE^−/−^ mice, which were fed with Western diet for 16 weeks. The results showed no significant differences except for mild difference of body weight at 12 weeks and 16 weeks.

We carried out gene expression chips in Sirt6 wild-type and Sirt6 homozygous knockout mouse embryonic fibroblasts (MEFs). Using the commercial Ingeniuty Pathway Analysis (IPA) software analysis, we found that atherosclerosis signaling pathway was the second one among the 12 top pathways influenced by Sirt6 knockout (Supplementary Table 3). H60b (histocompatibility 60b), one member of NKG2D ligand family whose upregulation exacerbated atherosclerosis development^17^, is the top one gene that changed in atherosclerotic pathway (Supplementary Table 4). Its expression was low in wild-type MEF, but profoundly increased by Sirt6 knockout (Supplementary Table 5). NKG2D ligands also include H60a, H60b, Rae-1α, Rae-1δ and Rae-1ε in mice^17^. H60a was also increased by Sirt6 knockout. However, H60a is a pseudogene in C57/BL6^18^. We found no significant change for Rae-1α, Rae-1δ and Rae-1ε.

**Figure 4:**
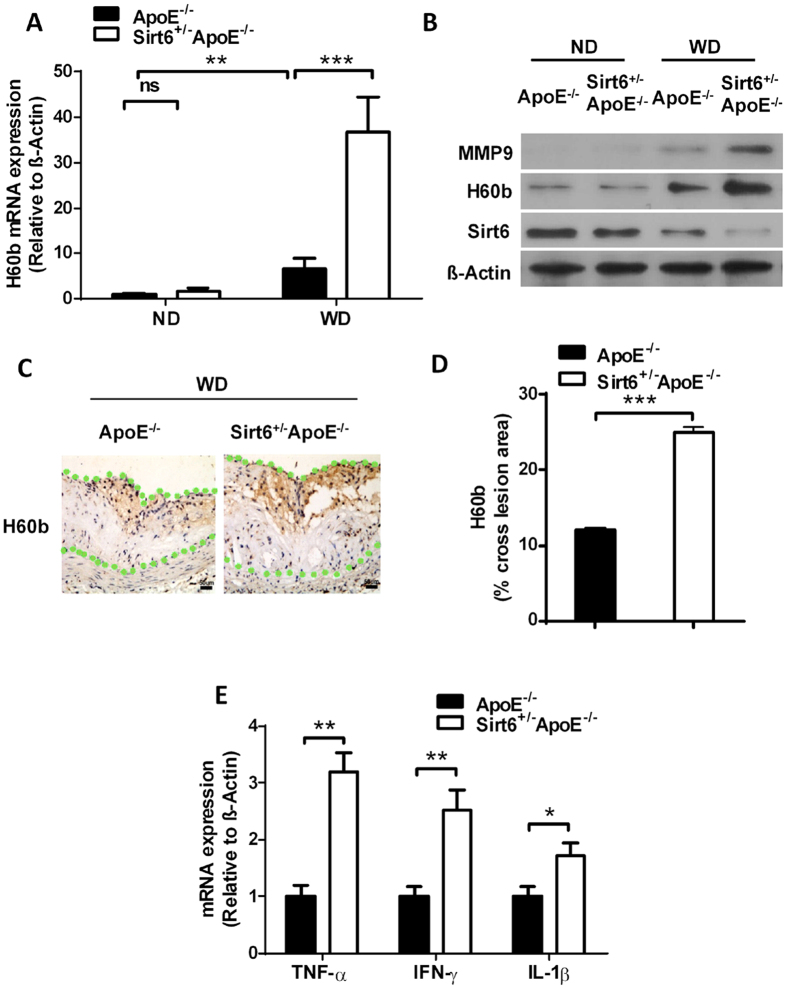
Sirt6 heterozygous mice exhibit increased expression of H60b. (**A**) The mRNA level of H60b in the aortas was determined (n = 4 each group). The expression level of H60b in aortas from ApoE^−/−^ mice on normal chow diet group normalized to β-Actin was set to 1. P value was obtained by two-way analysis of variance (ANOVA) plus a *post hoc* analysis using the Bonferroni test. ND: normal chow diet, WD: Western diet. (**B**) The protein level of H60b in the aortas was determined (n = 4 each group). (**C**) The H60b protein level in the atherosclerotic plaque was assessed by immunohistochemistry. (**D**) Quantitative analysis of the expression levels of Sirt6. The total cross lesion area is outlined with green dots and the positive signal was normalized to total cross lesion area. Student’s t test was used to calculate the P value. (**E**) The mRNA level of inflammation-associated cytokines in aortas of ApoE^−/−^ and Sirt6^+/−^ApoE^−/−^ fed Western diet for 16 weeks was determined (n = 4 each group). Student’s t test was used to calculate the P value. (*P < 0.05, **P < 0.01, ***P < 0.005).

NKG2D ligands are membrane proteins. Western diet increases NKG2D ligands and recruits NK/NKT cells, which express NKG2D receptor, and leads to expression of pro-atherosclerotic cytokines, including TNF-α, IFN-γ and IL-1β^17^,^19^,^20^. We examined whether Sirt6 regulated NKG2D ligand expression *in vivo*. We found that when mice were fed with normal chow diet, the expression level of H60b was low. Sirt6 heterozygosity had slight but not significant effect on H60b expression. H60b expression was higher when mice were fed with Western diet. Sirt6 heterozygosity further increased H60b expression, at mRNA level, as assessed by RT-PCR (Fig. 4A), and at the protein level, as assessed by western blot (Fig. 4B). Immunohistochemistry experiments showed that H60b expression was increased in Sirt6^+/−^ApoE^−/−^ atherosclerotic plaques (Fig. 4C,D). We found no significant increase for expression of Rae-1 members by Sirt6 heterozygosity, including Rae-1α, Rae-1δ and Rae-1ε (Supplementary Fig. 4). Concomitant with increased H60b expression, Sirt6 heterozygosity showed increased inflammatory molecules, including TNF-α, IFN-γ and IL-1β (Fig. 4E).

### Sirt6 suppresses NKG2D ligand expression in macrophages and endothelial cells

**Figure 5:**
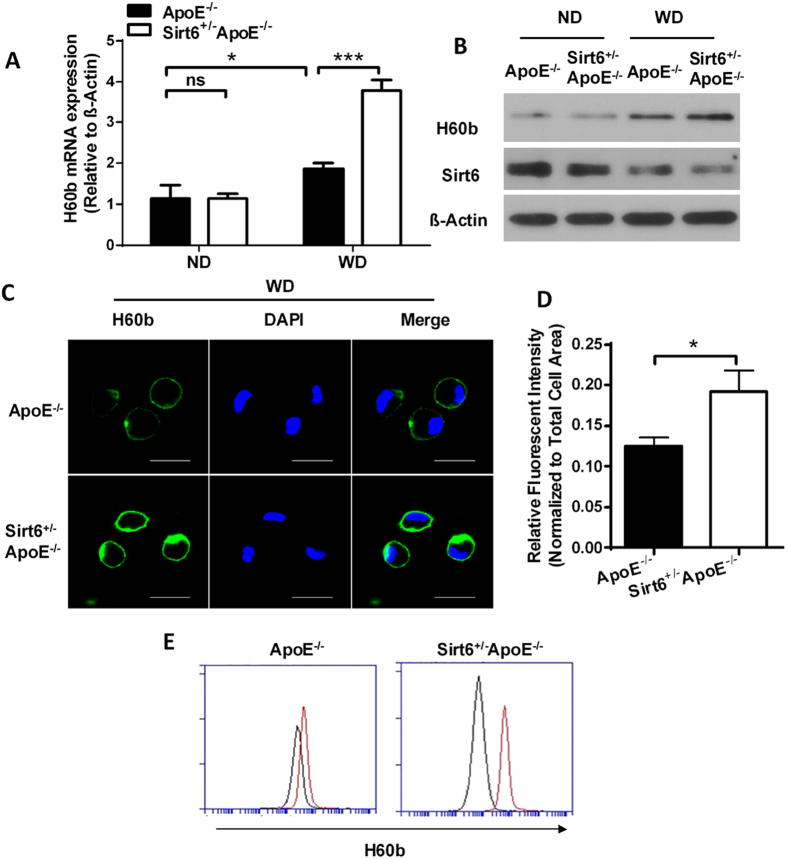
Sirt6 inhibits H60b expression in macrophages. (**A**) The mRNA level of H60b expression in macrophages from mice fed with normal chow diet (ND) or Western diet (WD) was determined. The expression level of H60b in macrophages from ApoE^−/−^ mice on normal chow diet group was normalized to β-Actin and was set to 1. P value was obtained by two-way analysis of variance (ANOVA) plus a *post hoc* analysis using the Bonferroni test. (n = 4 each group) (**B**) The protein level of H60b was determined similarly with (**A**) (n = 4 each group). (**C**) Immunofluorescence and confocal experiments were performed to determine H60b expression in macrophages from ApoE^−/−^ and Sirt6^+/−^ApoE^−/−^ mice fed Western diet for 16 weeks. (**D**) Densitometric analysis of the expression level of Sirt6. Student’s t test was used to calculate the P value. (**E**) Flow cytometry was performed to determine the number of H60b positive cells. The red curve indicates H60b positivity, and the blue curve indicates the negative control. (*P < 0.05, ***P < 0.005).

NKG2D ligand upregulation in macrophages and endothelial cells is important for exacerbating atherosclerosis development^17^,^19^. To further investigate the effects of Sirt6 on NKG2D ligand expression, we carried out experiments in these two types of cells. ApoE^−/−^ mice and Sirt6^+/−^ApoE^−/−^ mice were fed with either normal chow diet or Western diet, and peritoneal macrophages were isolated. Macrophages from mice fed with normal chow diet showed low level of H60b expression. Sirt6^+/−^ApoE^−/−^ group showed no significant increase than ApoE^−/−^ group. When both groups were fed with Western diet, the H60b expressions were increased. Sirt6^+/−^ApoE^−/−^ mice had higher level of H60b expression than ApoE^−/−^ mice (Fig. 5A,B). Immunofluorescence and flow cytometry analysis further revealed that the Sirt6^+/−^ApoE^−/−^ peritoneal macrophages had higher levels of H60b expression (Fig. 5C–E).

We next investigated the effects of Sirt6 on NKG2D ligand expression in endothelial cells. Because endothelial cells were difficult to obtain from mice, we utilized human umbilical vein endothelial cells (HUVECs). The major histocompatibility complex class I chain-related family members MICA and MICB are two NKG2D ligands in human. We observed that Sirt6 inhibited the expression of MICA/B. Sirt6 RNAi increased MICA/B expression (Supplementary Fig. 5A,B), while overexpression of Sirt6 decreased MICA/B expression (Supplementary Fig. 5C–E).

Taken together, these results indicate the suppressive roles of Sirt6 for NKG2D ligand expression.

### Upregulation of NKG2D ligand mediates increased cytokine expression of NK cells

**Figure 6:**
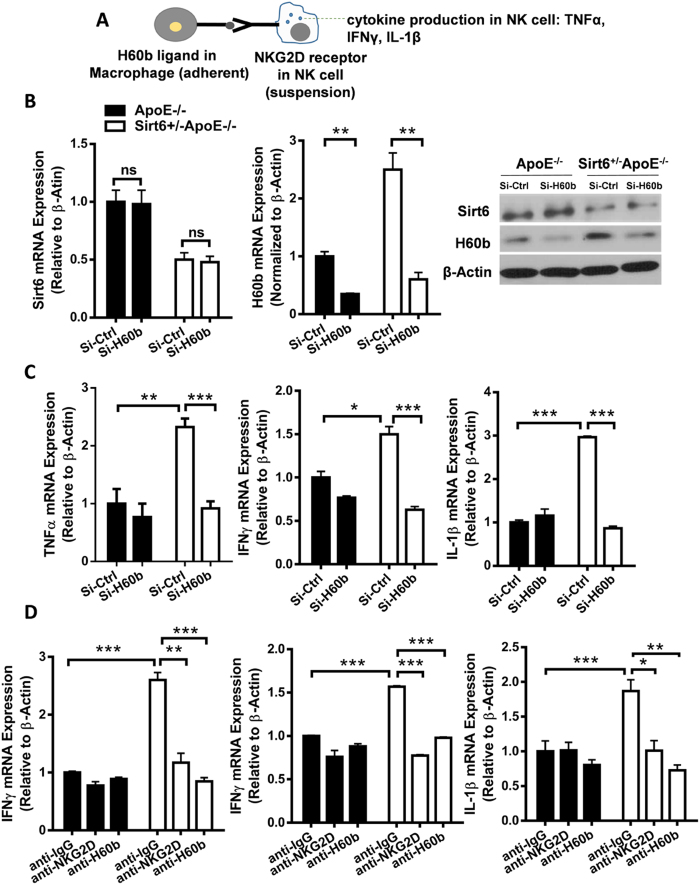
H60b mediates increased cytokine expression of NK Cells by Sirt6 heterozygosity. (**A**) Schematic diagram of the experiments. Peritoneal macrophage, isolated from ApoE^−/−^ and Sirt6^+/−^ApoE^−/−^ mice, were incubated to adhere to culture plate. Cells were treated with 30 μg/ml ox-LDL for 24 hours, and cocultured with NK cells, at 1:10 ratio for 6 hours. The NK cells were collected and used for determination of cytokine expression by realtime PCR. (**B,C**) Macrophages were transfected with Si-Ctrl or Si-H60b to knockdown H60b, cultured for 24 hours, treated with 30 μg/ml ox-LDL for 24 hours, and then co-incubated with NK cells at 1:10 ratio for 6 hours. (**B**) Macrophages were collected to determinate Sirt6 and H60b expression at the mRNA level and protein level. (**C**) The NK cells were collected and used for determination of cytokine expression by realtime PCR. (**D**) To block ligand-receptor interaction, isotype IgG antibody or NKG2D antibody (R&D, BAM1547, 3 μg/ml) or H60b antibody (R&D, MAB1155, 3 μg/ml) were added for 2 hours before co-incubation with NK cells. The NK cells were collected and used for determination of cytokine expression by realtime PCR. P value was obtained by two-way analysis of variance (ANOVA) plus a *post hoc* analysis using the Bonferroni test. (*P < 0.05, **P < 0.01, ***P < 0.005).

NKG2D ligand upregulation in macrophages/endothelial cells would activate NK/NKT cells, through ligand-receptor interaction, to express pro-atherosclerotic inflammatory molecules in NK/NKT cells^17^,^19^,^20^. To observe the effects of Sirt6 on NK cell activation and inflammation, and to investigate whether NKG2D ligand mediates this effect, we isolated macrophages from ApoE^−/−^ mice and Sirt6^+/−^ApoE^−/−^ mice, cocultured with NK cells, and determined cytokine expression in NK cells by realtime PCR (Fig. 6A). We found that Sirt6 heterozygosity in macrophages increased expression of cytokines in NK cells, including TNF-α, IFN-γ and IL-1β. When H60b expression was effectively decreased by H60b RNAi, a mild decrease of inflammatory gene expression was observed in wild-type group, while an obvious decrease of inflammatory gene expression was observed in heterozygous group. H60b knockdown almost completely antagonized increase of cytokine expression in Sirt6 heterozygous group, suggesting that H60b is necessary for mediating the effects of Sirt6 heterozygosity (Fig. 6B,C). We also used H60b antibody or NKG2D antibody to block H60b-NKG2D receptor interaction to observe the effects. The results showed that both H60b antibody and NKG2D antibody almost completely antagonized the effects of Sirt6 heterozygosity, further demonstrating the importance of H60b in mediating the effects of Sirt6 (Fig. 6D).

In HUVECs, inflammation in NK cells was also increased by Sirt6 downregulation in HUVECs. Addition of antibody for MICA/B and NKG2D receptor antagonized this effect (Supplementary Fig. 6).

### Sirt6 regulates acetylation levels of H3K9 and H3K56 in NKG2D ligand gene promoters

**Figure 7:**
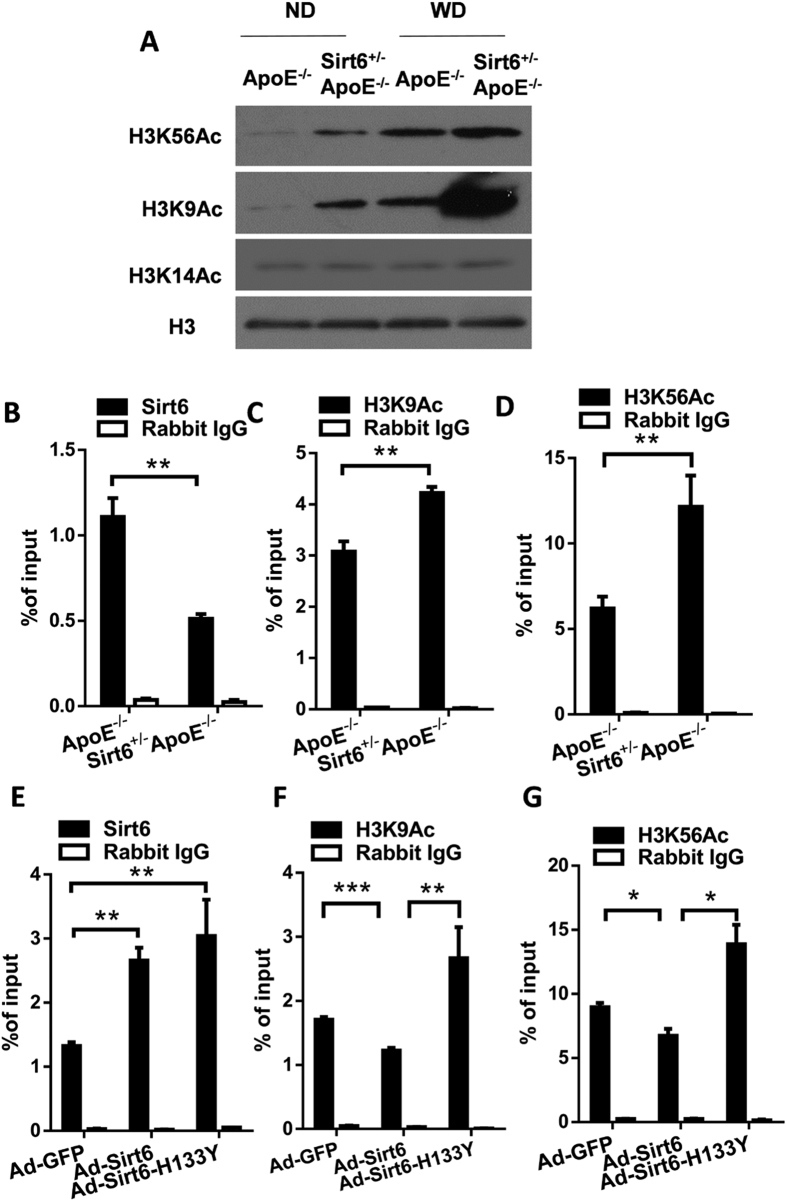
Sirt6 binds to H60b gene promoter and deacetylates H3K9 and H3K56. Peritoneal macrophages were isolated from ApoE^−/−^ and Sirt6^+/−^ApoE^−/−^ fed with Western diet for 16 weeks. (**A**) Total H3K9Ac and H3K56Ac levels were determined by western blot. (**B–D**) ChIP assays were performed to assess Sirt6 binding (**B**), H3K9Ac level (**C**), and H3K56Ac level (**D**) in the promoter of H60b (−158/−53bp). (**E–G**) Macrophages were infected with Ad-GFP, Ad-Sirt6 or Ad-Sirt6-H133Y, and ChIP assays were performed to assess Sirt6 binding (**E**), H3K9Ac level (**F**), and H3K56Ac level (**G**) in the H60b promoter (−158/−53bp). P value was obtained by two-way analysis of variance (ANOVA) plus a *post hoc* analysis using the Bonferroni test. (*P < 0.05, **P < 0.01, ***P < 0.005).

Sirt6 is an H3K9 deacetylase and H3K56 deacetylase^21^,^22^. We investigated whether Sirt6 modulated H3K9 and H3K56 acetylation levels in the promoters of NKG2D ligands. Immunohistochemistry experiments showed that in plaques, total H3K9 and H3K56 acetylation levels were increased by Sirt6 heterozygosity (Supplementary Fig. 7). Peritoneal macrophages from Sirt6^+/−^ApoE^−/−^ mice exhibited increased total H3K9 and H3K56 acetylation than that from ApoE^−/−^ mice (Fig. 7A). ChIP analysis revealed that Sirt6 binding to the promoter of H60b was decreased in heterozygous group, while H3K9 and H3K56 acetylation levels in H60b promoter were higher in heterozygous group than that in wild-type group (Fig. 7B–D). We further infected macrophages with Sirt6 wild-type or Sirt6 dominant-negative H133Y adenovirus^23^. The results showed that overexpressed Sirt6 was recruited to H60b promoters (Fig. 7E). Wild-type Sirt6 inhibited H3K9 and H3K56 acetylation levels, while H133Y Sirt6 lost this inhibitory function (Fig. 7F,G). These results indicate that Sirt6 binds to H60b promoter and decreases the acetylation levels of H3K9 and H3K56 of H60b promoter in macrophages. In HUVECs, we also observed an inhibitory effect of wild-type Sirt6 overexpression on total H3K9 and H3K56 acetylation (Supplementary Fig. 8A). Sirt6 bound to MICA and MICB promoters (Supplementary Fig. 8B) and regulated the H3K9 and H3K56 acetylation levels in a deacetylase-activity-dependent manner (Supplementary Fig. 8C–F). Taken together, these results indicate that Sirt6 regulates H3K9 and H3K56 acetylation levels of NKG2D ligand gene promoters.

## Discussion

Previous reports show that genetic variants of Sirt6 are associated with atherosclerosis^24^ and Sirt6 expression level is decreased in diabetic atherosclerosis^25^. Our results provide direct *in vivo* evidence that Sirt6 is involved in atherosclerosis development. Sirt6 heterozygous mice show exacerbated atherosclerosis and exhibit feature of instable atherosclerotic plaques than wild-type mice. Sirt6 heterozygosity shows increased NKG2D ligand expression in macrophages and endothelial cells, leading to NK cell activation and increased levels of inflammatory cytokines in NK cells. Importantly, NKG2D ligand knockdown by RNAi as well as antibody against H60b or NKG2D almost completely blocks this effect. Mechanistically, Sirt6 regulates H3K9 and H3K56 acetylation levels of NKG2D ligand promoters.

Plaque lesion size indicates the extent of atherosclerosis development. We show that Sirt6 heterozygosity increased plaque development by different methods, including IMT, Oil Red O staining area, H&E staining area and necrotic core area. In addition to plaque area, plaque stability is a more accurate predictor of plaque rupture and clinical events and stabilizing plaque is becoming an important focus for atherosclerosis treatment^15^,^16^. Here, we found that Sirt6 heterozygosity exhibits features of plaque instability, including larger lipid core, more infiltration of macrophage, less SMC and less collagen content. We used Sirt6 heterozygous knockout mice in this manuscript. We observed that plasma lipid or glucose profile is no significant difference between ApoE^−/−^ and Sirt6^+/−^ApoE^−/−^ mice. The observed phenotype is probably the combined effects of Sirt6 heterozygous knockout in different kinds of vascular cells, which include at least macrophages and endothelial cells. Our results provide evidence that downregulation of Sirt6 in macrophages and endothelial cells are important for NKG2D ligand upregulation and increased cytokine expression. Further experiment using Sirt6 conditional knockout in different types of vascular cells will further demonstrate the specific roles of Sirt6 in each cell type.

NKG2D ligand expression and NKG2D ligand-receptor interaction mediated innate immune activation are shown to play important roles in atherosclerotic plaque development^17^,^26^,^27^. Sirt6 is an H3K9 and H3K56 deacetylase. We provide evidence that Sirt6 epigenetically regulates NKG2D ligand expression and immune activation. In mice, NKG2D ligands include Rae-1 members (Rae-1α, Rae-1δ, Rae-1ε) and H60 members (H60a, H60b, H60c). Although NKG2D receptor knockout protects mice from atherosclerosis, it is more complex for the expressions and roles of NKG2D ligands. Some ligands, including H60b, Rae-1δ, Rae-1ε, are associated with exacerbated atherosclerosis and are reported to be increased in atherosclerotic plaques in ApoE^−/−^ C56BL/6 mice fed with diet containing 35.5% fat for 8 to 9 weeks^17^. On the other hand, Rae-1ε, which is also called Raet-1ε, is reported to be decreased in atherosclerotic plaques in ApoE^−/−^ FVB mice fed with AIN76a diet for 16 weeks, and transgenic mice of Rae-1ε show decreased atherosclerosis development^28^. So, the expression patterns and functions of NKG2D ligands may be diverse according to certain circumstances. In our experiments, we found that when ApoE^−/−^ mice on the background of C56BL/6 are fed with Western diet containing 10% fat and 1.25% cholesterol for 16 weeks, H60b expression, but not Rae-1 member expression, is induced by Sirt6 heterozygosity. H60b knockdown as well as antibody of H60b or NKG2D receptor antagonizes effects of Sirt6 heterozygosity, indicating that H60b is important for mediating Sirt6’s effect. Whether H60b or NKG2D knockout rescue the phenotype of Sirt6 heterozygosity *in vivo* needs further investigation.

In summary, our study demonstrates that heterozygous Sirt6 mice have more severe atherosclerosis and exhibit feature of less stable atherosclerotic plaques, suggesting the protective roles of Sirt6 in atherosclerosis. Sirt6 regulates H3K9 and H3K56 acetylation levels at the promoters of NKG2D ligands. The protective role of Sirt6 makes Sirt6 a potential target in preventing atherosclerosis.

## Methods

### Human atherosclerotic plaques and normal aortas

Human atherosclerotic plaques were obtained from patients undergoing carotid endarterectomy. Normal carotids were obtained from patients without coronary artery disease and stroke, and atherosclerotic plaques were excluded by morphological analysis. All experimental protocols were approved by the Ethical Committee of Chinese Academy of Medical Sciences and Peking Union Medical College, and conform to the principles outlined in the Declaration of Helsinki. The methods were carried out in accordance with the approved guidelines. The written informed consent was obtained from all subjects.

### Animal experiments

For animal experiments, all experimental protocols were approved by the Animal Care and Use Committee at the Institute of Basic Medical Sciences, Chinese Academy of Medical Sciences and Peking Union Medical College. The methods were carried out in accordance with the approved guidelines. The Sirt6 heterozygote (Sirt6^+/−^) mice on 129/SV background were originally obtained from the Jackson Laboratory (Bar Harbor, Me, USA) (Stock Number 006050). Sirt6 heterozygous mice (Sirt6^+/−^) were backcrossed with wild-type C57BL/6J mice for 8 generations and crossed with Apolipoprotein E-deficient (ApoE^−/−^) mice on a C57BL/6J background to get Sirt6^+/−^ ApoE^−/−^ mice and littermates. Then, acquired Sirt6^+/−^ApoE^−/−^ mice and littermates were fed with Western diet (containing 10% fat and 1.25% cholesterol), beginning at the age of 4 weeks and lasting for 16 weeks, to induce atherogenesis. Their plaques, blood cytokines, glucose and lipid levels, including total cholesterol, triglyceride, LDL cholesterol and HDL cholesterol, were then analyzed.

### Cell culture, plasmids, adenovirus generation and infection

Primary mouse embryonic fibroblasts (MEFs) were isolated from mouse embryos at embryonic day 13.5 (Ed), and cultured in DMEM medium (Gibco) with 10% fetal bovine serum (FBS) and penicillin/streptomycin. Mouse peritoneal macrophages were collected as described previously^29^ from the peritoneal cavity of ApoE^−/−^ or Sirt6^+/−^ApoE^−/−^ mice which had been injected intraperitoneally with 3% thioglycollate for 5 days. Human umbilical vein endothelial cells (HUVECs) were freshly isolated from human umbilical cord veins and cultured in M200 medium (Cascade Biologics, M-200-500) supplemented with low-serum growth supplement (Cascade Biologics, S-003-10) according to the manufacturer’s recommendations. The natural killer cells (NK cells) were isolated from mouse spleen. After mice were sacrificed by cervical dislocation, spleen were isolated and placed in cell culture dish. After adding the spleen cell isolation liquid (Dakewe Biotechnology, DKW33-R0100), the spleen were grinded gently. The dispersive spleen cells were through a filter and collected at 800g for 30 min. Then NK cells were isolated according to the manufacturer’s instructions (Miltenyi Biotechnology, 130-090-864). Mouse NK cells were cultured in α-MEM medium contained 2mM L-glutamine, 1.5 g/L sodium bicarbonate, 0.2 mM inositol, 0.1 mM 2-mercaptoethanol, 0.02 mM folic acid, 12.5% horse serum, 12.5% fetal bovine serum and 100–200 U/ml recombinant mouse IL-2 (Peprotech, 212-12). The human nature killer cell line NK92 cells (ATCC) were cultured in α-MEM medium containing the same components except replacing the recombinant mouse IL-2 with recombinant human IL-2 (Peprotech, 200-02).

The mouse Sirt6 expression plasmids were kind gifts from Dr. Frederic Van Gool^30^. The human Sirt6 siRNA sequence (Si-Sirt6)^31^ CCG GCT CTG CAC CGT GGC TAA was synthesized and the control sequence was acquired (Guangzhou RiBoBio Co). The Si-Sirt6 and the control Si-Ctrl were transfected into HUVECs with Lipofectamine 2000 (Invitrogen). The siRNA for mouse H60b was ordered from Oirgene (Trilencer-27 Mouse siRNA, SR407109). The Si-Ctrl or Si-H60b was transfected into macrophage by electroporation with Neon instrument. The parameters used were 1100 V, 20 ms, 2 pulses. The replication-defective adenoviral vectors expressing mouse Sirt6 (Ad-Sirt6), mouse Sirt6 H133Y (Ad-Sirt6-H133Y) and control green fluorescent protein (Ad-GFP) was constructed using AdEasy system^32^. Peritoneal macrophages and HUVECs were infected with adenovirus at MOI = 100 and used for further analyses.

### Ultrasound biomicroscopy measurements

The ultrasound imaging parameters of the ascending aortas were examined with a Vevo 770 Micro Ultrasound system (Visualsonics, Toronto, Canada). The mice were imaged by Ultrasound Biomicroscopy (UBM) at the level of the aortas. The plaque thickness and plaque area were determined using short-axis images of the ascending aortas^33^. The intima media thickness (IMT) and atherosclerotic lesions were readily visualized. During the experiments, light anesthesia was maintained with tribromoethanol (0.10–0.12 ml/10 g), which was injected intraperitoneally, resulting in a heart rate of approximately 300–400 beats/min.

### Analysis of atherosclerotic lesions and stability

Aortas, beginning outside the heart at the root and stopping at the iliac artery, were isolated from atherosclerotic mice. The samples were opened longitudinally, fixed in 4% paraformaldehyde and stained with Oil red O dye. The lesion-stained aortas were analyzed using Image Pro Plus 6.0 (IPP6) software. The histomorphometric characteristics of the plaques were analyzed by hematoxylin/eosin (H&E) staining. Additional staining methods included Oil red O dye, Masson’s trichrome staining (collagen), anti-α-smooth muscle actin antibody staining (smooth muscle cells, SMCs) and anti-Mac3 antibody staining (macrophages). Plaque stability was evaluated by comparing the percentages of the above mentioned plaque components in the entire plaque. The histological plaque stability score was calculated as follows^34^: plaque stability score = (SMC area + collagen area)/(macrophage area + lipid area).

### Immunohistochemical staining

The upper parts of the hearts containing the aorta root were harvested, fixed in 4% paraformaldehyde and paraffin-embedded. Sections of the aorta sinus in the aorta root (5-μm thick) were created and used for H&E staining or immunohistochemical staining. For H&E staining, the plaque areas of sections were stained by H&E staining and calculated in a blinded manner by using IPP6 software. For immunohistochemical staining, the sections were deparaffinized, and quenched with 3% (vol./vol.) hydrogen peroxide to remove endogenous peroxidase activity. Nonspecific binding were blocked in 10% goat serum in PBS at room temperature for 1 hour, then slides were incubated at 4 °C overnight with primary antibodies: α-SMA (Abcam, ab5694, 1:400), Mac3 (BD Pharmingen, 550292, 1:300), H60b (Santa Cruz Biotechnology, sc-20330, 1:100), H3K9Ac (Abcam, ab4441, 1:200), H3K56Ac (Epigentek, A-4026, 1:200). The slides were incubated with biotinylated secondary antibodies at 37 °C for 30 minutes and subsequently with horseradish peroxidase-labeled streptavidin solution at 37 °C for 20 minutes. Slides were then stained with diaminobenzidine (DAB kit, Vector Laboratories) and counterstained with hematoxylin.

### Immunofluorescence

Macrophages isolated from the ApoE^−/−^ mice or Sirt6^+/−^ApoE^−/−^ mice were cultured in the 24 well plates which were laid with sterile coverslip. After the cells grew well on the coverslip, the medium was removed and cells were washed with PBS. Cells were fixed gently with 4% paraformaldehyde for 10 minutes, treated with 0.2% Triton-X100 for 5 min to enhance the membrane permeability, blocked with 3% BSA solution for one hour, incubated with primary antibody against H60b (Santa Cruz Biotechnology, sc-20330, 1:50), at 4 °C overnight, and incubated with fluorescein-conjugated secondary antibody (Invitrogen, A-11078; 1:500) for one hour. The nuclei were visualized by stained with 4, 6-diamidino-2-phenylindole. The slides were washed and covered with mounting medium and fluorescence signal was detected in confocal microscopy.

### Flow cytometry

Macrophages were processed and stained with the monoclonal anti-H60b antibody (R&D, MAB1155, 3 μg/ml). The antibody was conjugated to a fluorochrome using the Lightning-link^®^ RPE Conjugation kit, and the appropriate IgG isotype controls were used (eBioscience). Flow cytometry was performed with an Accuri C6 Flow Cytometer (BD Biosciences), and the data were analyzed using C6 Flow Cytometer software.

### Cytokine expression in NK cells

Peritoneal macrophages isolated from ApoE^−/−^ and Sirt6^+/−^ApoE^−/−^ mice were incubated to adhere to culture plate. Cells were treated with 30 μg/ml ox-LDL (Beijing Xiesheng Biotech Co) for 24 hours. NK cells were isolated from mouse spleen. Adherent macrophages were then cocultured with suspended NK cells at 1:10 ratio for 6 hours. The NK cells were then collected and used for determination of intracellular cytokine expression by realtime PCR, including TNF-α, IFN-γ and IL-1β. The adherent macrophages were collected to determine Sirt6 and H60b expression. To knockdown H60b, macrophages were transfected with Si-Control or Si-H60b by Neon instrument with parameters of 1100 V, 20 ms, 2pulses. Cells were cultured for 24 hours before treatment with ox-LDL. To block ligand-receptor interaction, isotype IgG antibody or NKG2D antibody (R&D, BAM1547, 3 μg/ml) or H60b antibody (R&D, MAB1155, 3 μg/ml) were added for 2 hours before co-incubation with NK cells.

HUVECs were cultured, treated with 30 μg/ml ox-LDL for 24 hours, then cocultured with suspended NK92 cells at 1:10 ratio for 6 hours. The NK92 cells (ATCC) were collected and used for determination of cytokine expression by realtime PCR. To block ligand-receptor interaction, isotype IgG antibody or NKG2D antibody (R&D, BAM1547, 3 μg/ml) or MICA/B antibody (R&D, MAB13001, 3 μg/ml) were added for 2 hours before co-incubation with NK92 cells.

### Western blot

The antibodies used were as follows: Sirt6 (Abcam, ab62739, 1:1000; CST, 2590, 1:1000), H60b (Santa Cruz Biotechnology, sc-20330, 1:300), β-actin (Sigma, A5376, 1:5000), H3 (Abcam, ab1791, 1:1000), and H3K9Ac (Abcam, ab4441, 1:1000), H3K56Ac (Epigentek, A-4026, 1:1000), H3K14Ac (Abcam, ab52946, 1:1000), MICA/B antibody (R&D, MAB13001, 1:500).

### Chromatin immunoprecipitation (ChIP) assay

Cells were collected, fixed and cell nuclei were isolated and sonicated. After pre-clearing, the supernatant were immunoprecipitated using normal rabbit IgG (Santa Cruz Biotechnology, sc-2027), Sirt6 antibody (Abcam, ab62739), H3K9Ac antibody (Abcam, ab4441), or H3K56Ac antibody (Epigentek, A-4026). The immunoprecipitated DNA were PCR amplified using indicated primers in Supplementary Table 7.

### Microarray experiment

Wild-type (Sirt6^+/+^) and Sirt6 knockout (Sirt6^−/−^) MEFs were isolated and cultured. For each group, three independent mouse embryo fibroblast (MEF) preparations were mixed together for RNA extraction and Affymetrix microarray experiment. The data were analyzed by commercial Ingeniuty Pathway Analysis (IPA) software.

### Statistical analysis

The data are presented as means ± SEM. Statistical analyses were performed using Student’s t-test or ANOVA plus a *post hoc* analysis using the Bonferroni test, with significance set at p < 0.05.

## Additional Information

**How to cite this article**: Zhang, Z.-Q. *et al.* Epigenetic regulation of NKG2D ligands is involved in exacerbated atherosclerosis development in Sirt6 heterozygous mice. *Sci. Rep.* **6**, 23912; doi: 10.1038/srep23912 (2016).

## Supplementary Material

Supplementary Information
