# Supplementary material for: Epigenetic regulation of NKG2D ligands is involved in exacerbated atherosclerosis development in Sirt6 heterozygous mice
Source: Sci Rep. 2016 Apr 5;6:23912. doi: 10.1038/srep23912 (PMC4820703; doi:10.1038/srep23912)
Supplement: Supplementary Information [file srep23912-s1.pdf]

Supplementary Information

Title

Epigenetic regulation of NKG2D ligands is involved in exacerbated atherosclerosis development in Sirt6 heterozygous mice

Authors

Zhu-Qin Zhang[#], Si-Chong Ren[#], Ying Tan[#], Zuo-Zhi Li, Xiao-Qiang Tang, Ting-Ting Wang, De-Long Hao, Xiang Zhao, Hou-Zao Chen[*], De-Pei Liu[*]

From State Key Laboratory of Medical Molecular Biology, Department of Biochemistry and Molecular Biology, Institute of Basic Medical Sciences, Chinese Academy of Medical Sciences and Peking Union Medical College, No.5 Dong Dan San Tiao, Beijing 100005, P.R. China.

[#] These authors contribute equally to this work.

Corresponding authors

* Correspondence to Hou-Zao Chen, or De-Pei Liu. Tel: 86-10-69156415. Fax: 86-10-65105093. Email addresses: chenhouzao@ibms.cams.cn, liudp@pumc.edu.cn.

Supplementary tables

Supplementary Table 1. Blood pressure and heart rate in ApoE$^{-/-}$ mice and Sirt6$^{+/-}$ApoE$^{-/-}$ mice which were fed with Western diet for 16 weeks.

	ApoE$^{-/-}$ (n=11)	Sirt6$^{+/-}$ApoE$^{-/-}$ (n=10)	P value
HR (beats/min)	602 ± 17	620 ± 12	0.41
SBP (mmHg)	118 ± 8	117 ± 4	0.91
MBP (mmHg)	66 ± 4	69 ± 4	0.68
DBP (mmHg)	81 ± 4	84 ± 4	0.51

Student’s t test was applied to calculate the P value.

Supplementary Table 2. Levels of plasma lipids in $ApoE^{-/-}$ mice and $Sirt6^{+/-}ApoE^{-/-}$ mice which were fed with Western diet for 16 weeks.

	$ApoE^{-/-}$ (n=15)	$Sirt6^{+/-}ApoE^{-/-}$ (n=11)	P value
Total cholesterol (mmol/L)	24.21 ± 1.42	23.27 ± 2.32	0.74
Triglyceride (mmol/L)	0.13 ± 0.03	0.14 ± 0.04	0.86
HDL cholesterol (mmol/L)	0.38 ± 0.05	0.40 ± 0.06	0.79
LDL cholesterol (mmol/L)	7.96 ± 0.64	7.45 ± 0.81	0.63
Glucose (mmol/L)	5.60 ± 0.65	5.00 ± 0.73	0.27

Student's t test was applied to calculate the P value.

Supplementary Table 3. Top pathways affected by Sirt6 kncokout.

Ingenuity Canonical Pathways	-log(p-value)	Ratio
Hepatic Fibrosis / Hepatic Stellate Cell Activation	8.45E00	1.32E-01
Atherosclerosis Signaling	5.36E00	1.3E-01
Axonal Guidance Signaling	5.66E00	8.1E-02
Sertoli Cell-Sertoli Cell Junction Signaling	4.92E00	1.06E-01
Agranulocyte Adhesion and Diapedesis	4.58E00	1.01E-01
Granulocyte Adhesion and Diapedesis	4.44E00	1.02E-01
Role of Tissue Factor in Cancer	4.02E00	1.18E-01
Reelin Signaling in Neurons	3.47E00	1.27E-01
Leukocyte Extravasation Signaling	3.34E00	8.59E-02
Inhibition of Angiogenesis by TSP1	3.04E00	1.76E-01
Dendritic Cell Maturation	2.91E00	8.38E-02
Role of NFAT in Cardiac Hypertrophy	2.91E00	8.38E-02

Supplementary Table 4. Genes involed in atherosclerosis pathway.

Symbol	Entrez Gene Name	Affymetrix	Fold Change (ko/wt, log2)
H60b	H60b mRNA for histocompatibility-60b	1440145_at	5.96
LPL	lipoprotein lipase	1431056_a_at	2.231
ICAM1	intercellular adhesion molecule 1	1424067_at	2.325
IL6	interleukin 6	1450297_at	2.359
MMP9	matrix metallopeptidase 9 (gelatinase B, 92kDa gelatinase, 92kDa type IV collagenase)	1416298_at	2.544
APOD	apolipoprotein D	1416371_at	2.777
IL1RN	interleukin 1 receptor antagonist	1425663_at	2.796
ITGA4	integrin, alpha 4 (antigen CD49D, alpha 4 subunit of VLA-4 receptor)	1456498_at	2.989
VCAM1	vascular cell adhesion molecule 1	1436003_at	2.999
COL1A2	collagen, type I, alpha 2	1446326_at	-2.454
PDGFD	platelet derived growth factor D	1426319_at	-3.051
MMP3	matrix metallopeptidase 3 (stromelysin 1, progelatinase)	1418945_at	-3.142
SELP	selectin P (granule membrane protein 140kDa, antigen CD62)	1420558_at	-3.244
CLU	clusterin	1437689_x_at	-3.367

Supplementary Table 5. Expression of NKG2D ligands in MEF (selected from Supplementary Microarray Data).

Gene Symbol	Ratio (Sirt6$^{-/-}$ vs. Sirt6$^{+/+}$)	Sirt6$^{-/-}$_ Signal	Sirt6$^{-/-}$_ Detection	Sirt6$^{+/+}$_ Signal	Sirt6$^{+/+}$_ Detection
H60a	46.1984	905.7	P	2.3	A
H60b	5.96336	256.9	P	11.7	A
Rae1a/Rae1b/Rae1c/Rae1d /Rae1e	0.831352	395.3	P	532	P

"P": indicate presence. "A": indicates absence.

Supplementary Table 6. Primers used for realtime PCR**.**

**Primer name**	**Primer sequence**
Mouse Sirt6 Forward	CCTGGTCAGCCAGAACGTAG
Mouse Sirt6 Reverse	TACTGCGTCTTACACTTGGGA
Human Sirt6 Forward	CCAAGTTCGACACCACCTTT
Human Sirt6 Reverse	GGCACATTCTTCCACAAACA
Mouse TNF-α Forward	CCCCAAAGGGATGAGAAGTT
Mouse TNF-α Reverse	CACTTGGTGGTTTGCTACGA
Mouse IFN-γ Forward	CACGGCACAGTCATTGAAAG
Mouse IFN-γ Reverse	GTCACCATCCTTTTGCCAGT
Mouse IL-1β Forward	TACAGGCTCCGAGATGAACA
Mouse IL-1β Reverse	AGGCCACAGGTATTTTGTCG
Mouse H60b Forward	GCTGCCTCAACAAATTGTCA
Mouse H60b Reverse	CAGACCCTGGGTGTCAGAAT
Mouse Rae-lα Forward	GGGGAATGTTTGACACAACC
Mouse Rae-lα Reverse	CCCTGGCTTTGCAGATAAAT
Mouse Rae-lδ Forward	AGCTATGGATACACCAACGGG
Mouse Rae-lδ Reverse	ACGAAGCACTTCACTTCATCTG
Mouse Rae-lε Forward	GACCAAGCGCCATCATTTTAT
Mouse Rae-lε Reverse	AGCACTTCACGTCACACCAG
Human MICA Forward	TAGAATCCGGCGTAGTCCTG
Human MICA Reverse	CTGCATGTCACGGTGATGTT

Human MICB Forward	GTGGCCATCAGGAGAACAGT
Human MICB Reverse	GACGCCAGGTCAGTGTGATA
Mouse β-actin Forward	GGCTGTATTCCCCTCCATCG
Mouse β-actin Reverse	CCAGTTGGTAACAATGCCATGT
Human β-actin Forward	CTCTTCCAGCCTTCCTTCCT
Human β-actin Reverse	AGCACTGTGTTGGCGTACAG

Supplementary Table 7. Primers Used in ChIP

Primer name	Primer sequence
mH60b promoter Forward (-158/-53bp)	GTGGTGCACGCCTTTAATTC
mH60b promoter Reverse (-158/-53bp)	CTCTGTGGGCAGGGGATG
hMICA promoter Forward (-112/+34bp)	CCCCAGTTTCATTGGATGAG
hMICA promoter Reverse (-112/+34bp)	CAGCCAGAAGCAGAAAGACC
hMICB promoter Forward (-139/-15bp)	CTAAGTTCCGGGCCTCAGTT
hMICB promoter Reverse (-139/-15bp)	CTACGTCGCCACCTTCTCAG

Supplementary Figures and Figure Legends

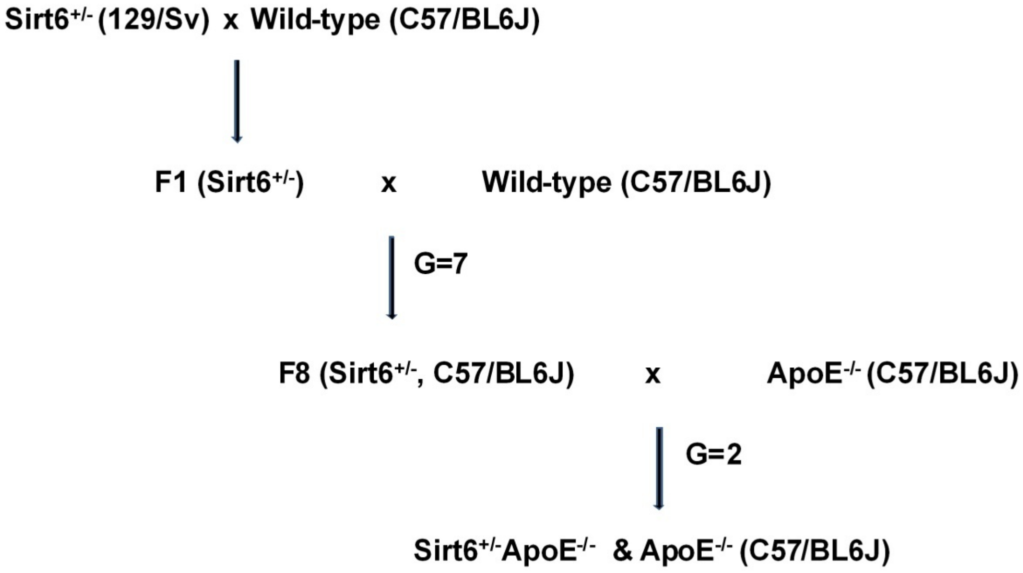

Supplementary Figure 1. Generation of Sirt6$^{+/-}$ApoE$^{-/-}$ mice.

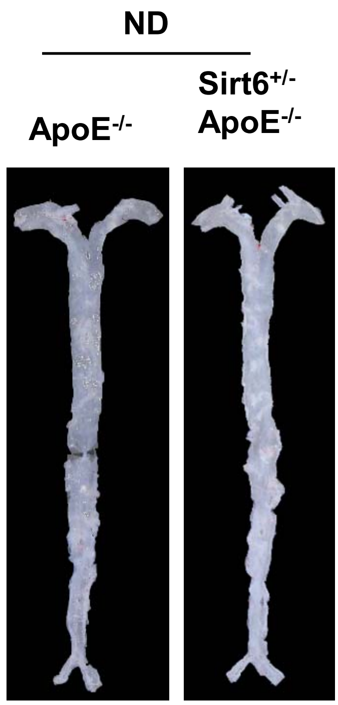

Supplementary Figure 2. Representative photographs showing Oil Red O staining of aortas of ApoE$^{-/-}$ mice and Sirt6$^{+/-}$ApoE$^{-/-}$mice (n=5 each group) fed with normal chow diet (ND).

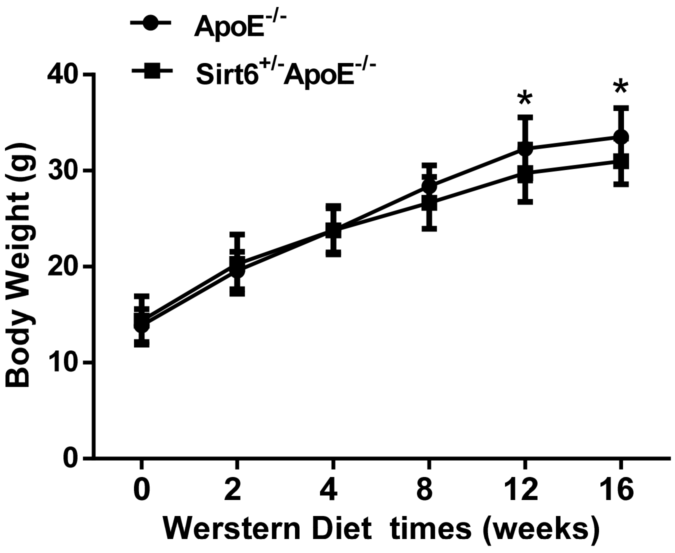

Supplementary Figure 3. Body weight of ApoE$^{-/-}$ mice and Sirt6$^{+/-}$ApoE$^{-/-}$ mice which were fed with Western diet for 16 weeks (n=15 each group at each time point). P value was obtained by two-way analysis of variance (ANOVA) plus a *post hoc* analysis using the Bonferroni test. (*P<0.05)

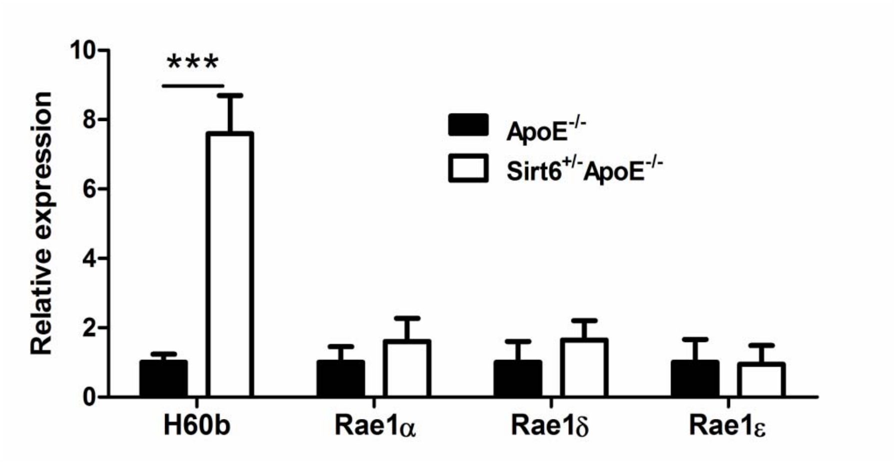

Supplementary Figure 4. ApoE$^{-/-}$ mice and Sirt6$^{+/-}$ApoE$^{-/-}$ were fed with Western diet for 16 weeks. Expression levels for NKG2D ligands in aortas were determined (n=4). Student's t test was applied to calculate the P value. (***P<0.005)

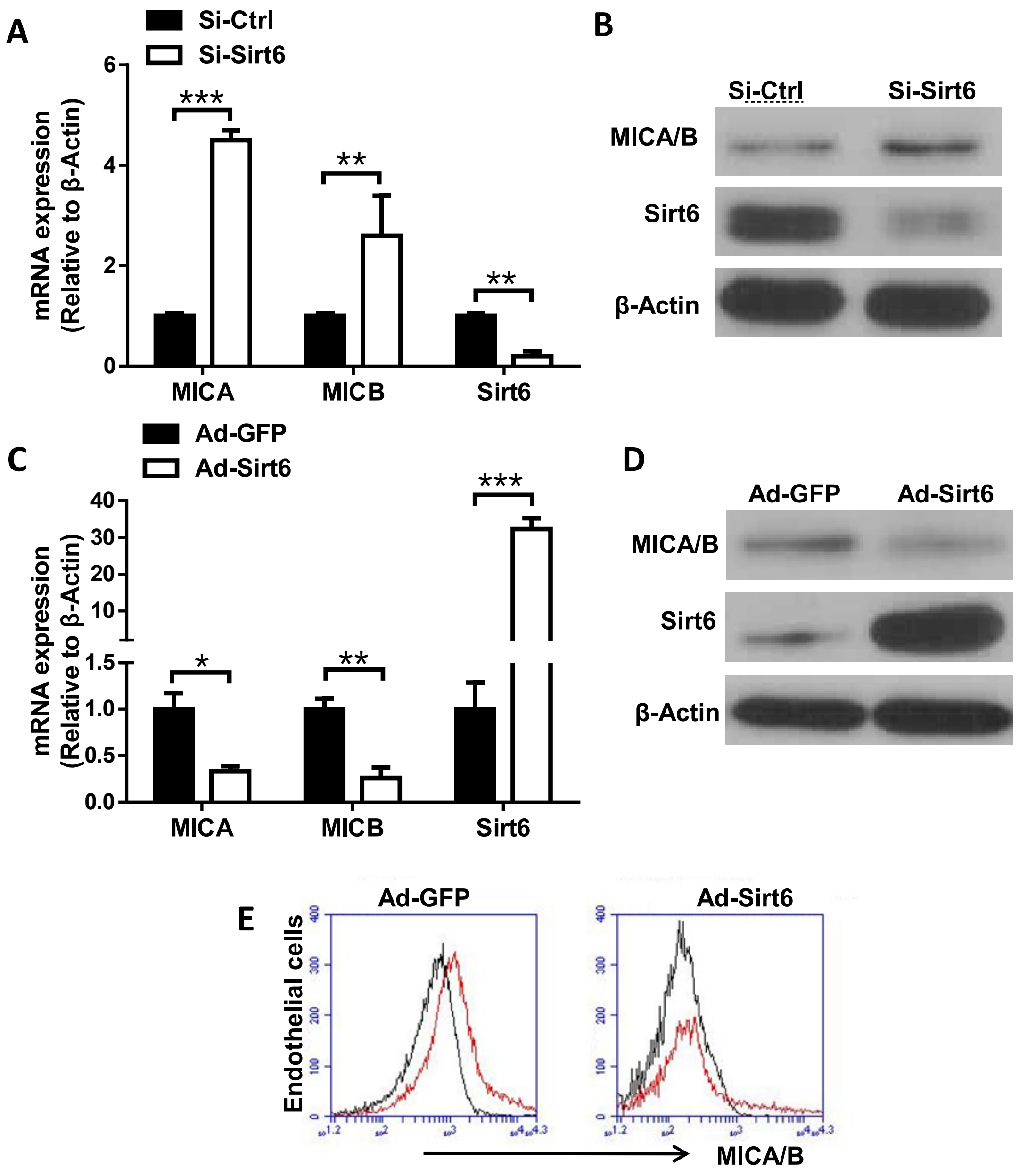

Supplementary Figure 5. Sirt6 inhibits MICA/B ligands expression in HUVECs. (A-B) HUVECs were transfected with Si-Ctrl or Si-Sirt6 and mRNA (A) and protein (B) levels of MICA/B were determined. (C-D) HUVECs were infected with Ad-GFP or Ad-Sirt6, mRNA(C) and protein (D) levels of MICA/B were determined. (E) HUVECs were infected with Ad-GFP or Ad-Sirt6, MICA/B quantity was determined by flow cytometry. Student's t test was applied to calculate the P value. (*P<0.05,

**P<0.01, ***P<0.005)

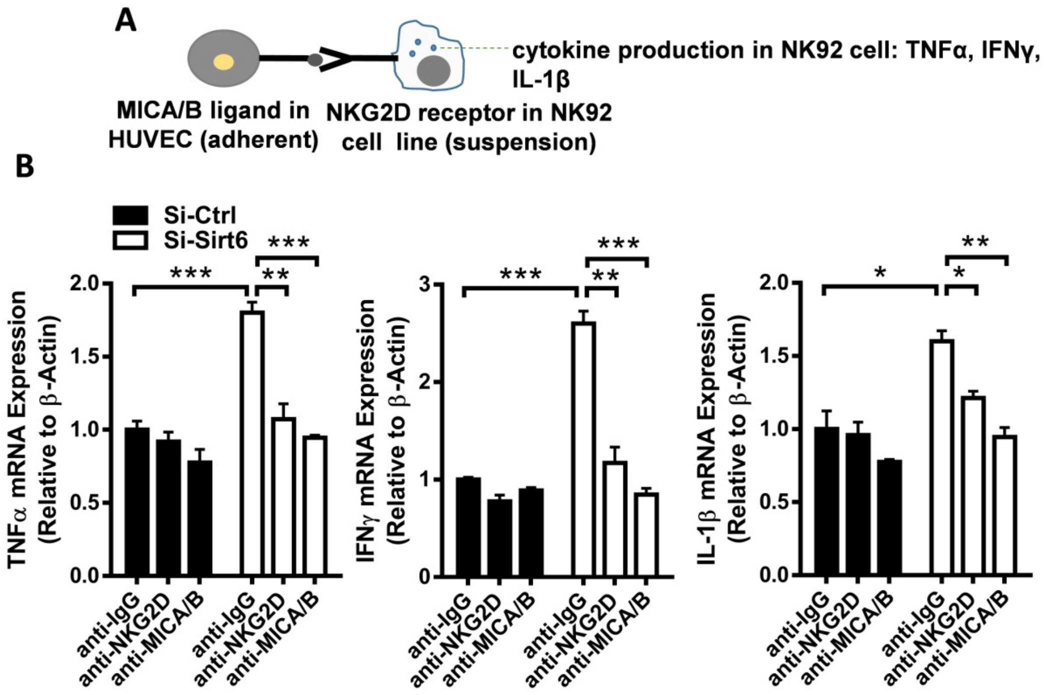

Supplementary Figure 6. MICA/B mediates increased cytokine expression in NK92 cell by Sirt6 downregulation. (A) Schematic diagram of the experimental process. Adherent HUVECs were transfected with Si-Ctrl or Si-Sirt6. 24 hours later, cells were treated with 30 μg/ml ox-LDL for 24 hours, then cocultured with NK92 cells at 1:10 ratio for 6 hours. The NK92 cells were collected and used for determination of cytokine expression by realtime PCR. (B) Experiments were performed as in (A). To block ligand-receptor interaction, isotype IgG antibody or NKG2D antibody (R&D, BAM1547, 3 μg/ml) or MICA/B antibody (R&D, MAB13001, 3 μg/ml) were added for 2 hours before co-incubation with NK92 cells. P value was obtained by two-way analysis of variance (ANOVA) plus a *post hoc* analysis using the Bonferroni test. (*P<0.05,**P<0.01, ***P<0.001)

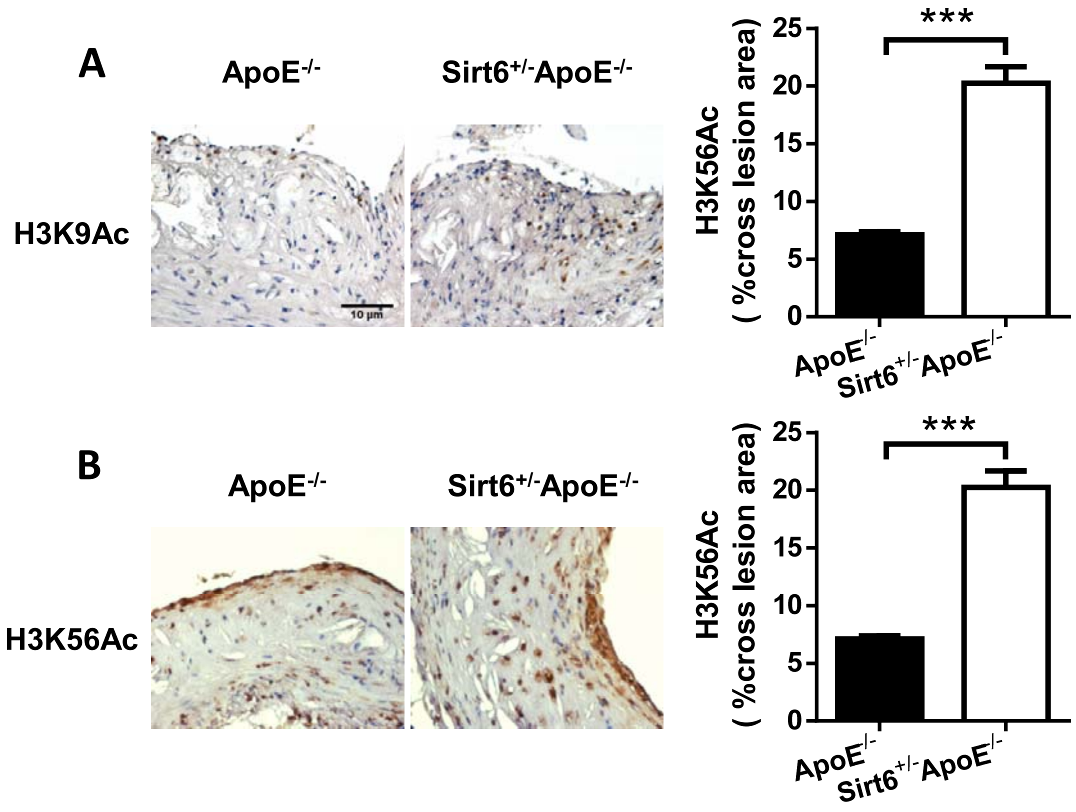

Supplementary Figure 7. H3K9Ac and H3K56Ac levels were increased in Sirt6 heterozygous atherosclerotic plaques. (A-B) Representative immunostaining was performed to detect H3K9Ac (A) and H3K56Ac (B) levels in atherosclerotic plaques of ApoE$^{-/-}$ mice and Sirt6$^{+/-}$ApoE$^{-/-}$ mice fed with Western diet for 16 weeks. The indicated area was normalized to total cross lesion area and the statistical analysis is shown on the right side (n=6 each group). Student's t test was used to calculate the P value in A and B. (***$P<0.005$)

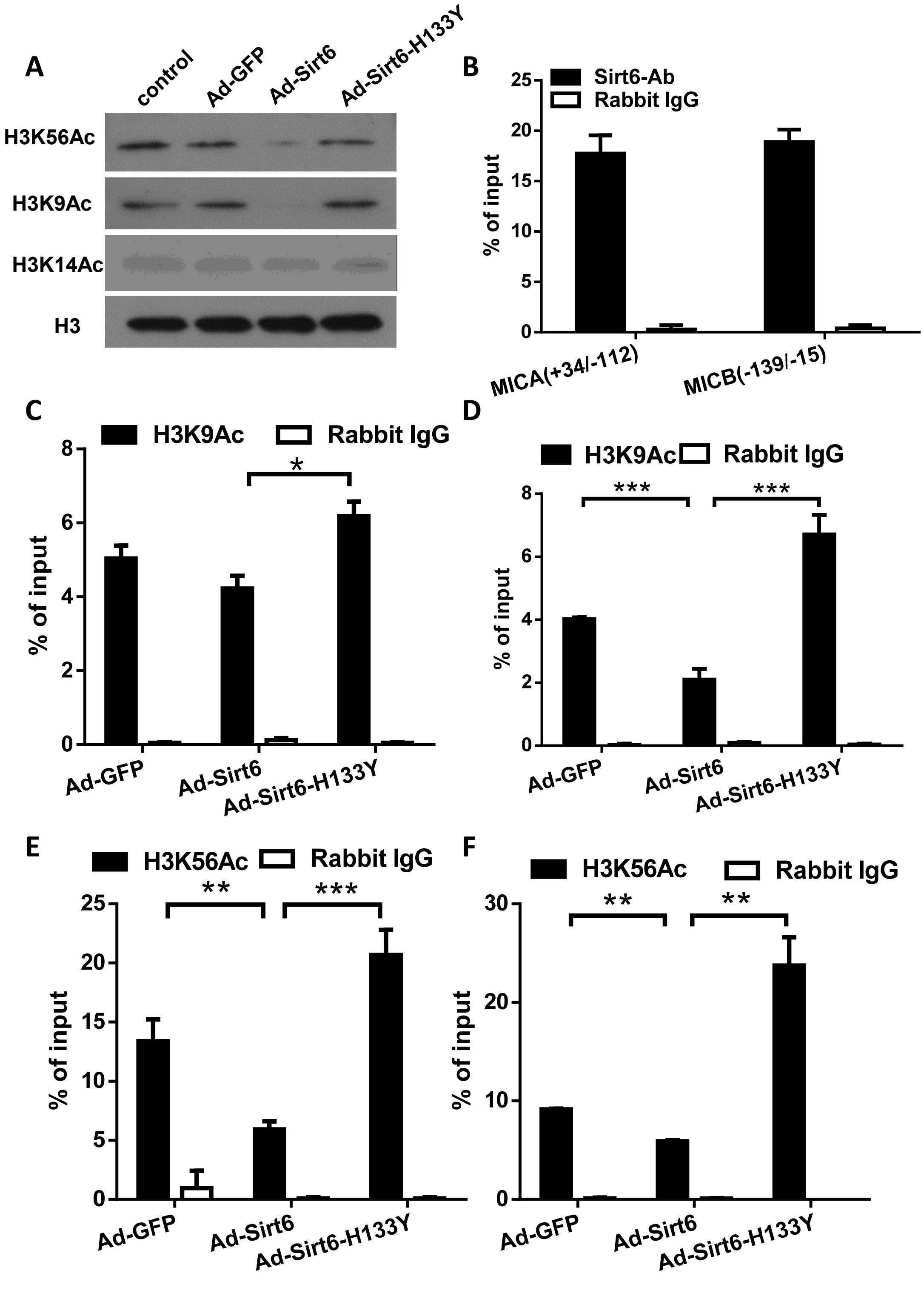

Supplementary Figure 8. Sirt6 binds to the promoters of MICA/B genes and deacetylates H3K9 and H3K56. (A) Total H3K9Ac and H3K56Ac in Ad-GFP, Ad-Sirt6 and Ad-Sirt6-H133Y infected HUVECs. (B) ChIP assays for Sirt6 binding to

the MICA/B promoters in HUVECs. (C-F) HUVECs were infected with Ad-GFP, Ad-Sirt6 or Ad-Sirt6-H133Y. The H3K9Ac levels in MICA (C) and MICB (D) and H3K56Ac levels in MICA (E) and MICB (F) gene promoters were determined by ChIP assays. P value was obtained by two-way analysis of variance (ANOVA) plus a *post hoc* analysis using the Bonferroni test. (*P<0.05, **P<0.01, ***P<0.001)
